# Factors contributing to the sharing of COVID-19 health information amongst refugee communities in a regional area of Australia: a qualitative study

**DOI:** 10.1186/s12889-022-13850-1

**Published:** 2022-07-28

**Authors:** Sunita Joann Rebecca Healey, Nafiseh Ghafournia, Peter D. Massey, Karinne Andrich, Joy Harrison, Kathryn Taylor, Katarzyna Bolsewicz

**Affiliations:** 1 Multicultural and Refugee Health Services, HNE Health, Harker Building, Wallsend Health Services, Longworth Ave, Wallsend NSW, Newcastle, 2287 Australia; 2 College of Medicine & Dentistry, James Cook University, Townsville, QLD 4811 Australia; 3 Multicultural and Refugee Health Services, HNE Health, Armidale Community Health Centre, 226 Rusden St, Armidale, NSW 2350 Australia; 4 Central Coast Population Health Unit, Level 1, 4 Watt St, Gosford, NSW 2250 Australia; 5 The University of Newcastle, University Drive, Callaghan NSW, Newcastle, 2308 Australia

**Keywords:** Refugees, Cultural diversity, COVID-19, Health literacy, Trust, Information dissemination, Social cohesion, Australia

## Abstract

**Background:**

The COVID-19 pandemic has had a disproportionate impact on culturally and linguistically diverse (CALD) groups worldwide. Newly emerging CALD populations formed by recently arrived refugees are predisposed to even greater health disadvantages due to complexities of the refugee experience. The aim of this study was to explore how culture, refugee experiences and existing relationships shaped what COVID-19 messages were listened to and shared during the early-mid phases of the pandemic. The work focused on three newly emerging refugee groups in the Hunter New England region, Australia: Afghan, Congolese and Syrian communities.

**Methods:**

Qualitative, semi-structured interviews were conducted to explore the experiences and stories of 15 adult community members, nine influential members and six service providers. All community members arrived in Australia on or after January 2014. Interpreter-assisted interviews were conducted with small groups or individuals, audio-recorded and transcribed verbatim in English. Three levels of thematic data analysis were employed to uncover the important issues and experiences of the participants.

**Results:**

Three key themes and several subthemes were identified. The themes were: 1) Experience as a refugee uniquely influences COVID-19 message communication; 2) Refugee groups use diverse practices when accessing and sharing COVID-19 messages; and 3) Official government messages could be improved by listening and tailoring to community needs.

**Conclusions:**

Effective health messaging relies on reaching communities in a culturally acceptable and meaningful way. Official COVID-19 messages can be tailored to engage newly emerging communities by improving the quality of the content, delivery and format whilst working collaboratively with communities and trusted service providers. Further mutual research is needed to understand emerging communities’ viewpoints. The use of culturally informed approaches is recommended.

## Background

In many countries the COVID-19 pandemic has exacerbated pre-existing health disparities based on ethnicity, resulting in higher risks of disease and death among immigrants [1]. Newly emerging culturally and linguistically diverse (CALD) populations are a group where even greater health disparities occur. These communities may arise when people migrate due to forced displacement or other significant events. Many resettled refugees face significant health disadvantage compared with non-refugee immigrants in their new country [2]. Some of the factors leading to the ongoing health disadvantage and risk of exacerbation include the limited education opportunities, past experiences of trauma and competing priorities [3, 4]. These factors further contribute to inequitable access to healthcare, including health information access [5].

There are a range of government and non-government programs supporting newly arrived refugees in Australia. The Australian Humanitarian Settlement Program (HSP) provides support for life skills, such as education and employment [6]. Free unlimited English classes are available for eligible adults with low English levels, until vocational English is achieved [7]. Health and schooling are provided primarily through state government. Non-government services include settlement agencies, counselling services and advocacy groups [8]. Temporary housing and welfare packages are provided by settlement agencies with federal HSP government funding, along with case management and linkages to services such as Education, Health and Social Services [9]. But these support services are not enough to prevent all disadvantage, especially during a rapidly evolving pandemic.

Refugees often struggle to access culturally and linguistically appropriate information about COVID-19 [10]. Background experiences related to dislocated education and post-traumatic stress disorder may impair refugees’ ability to understand COVID-19 messages [11]. Moreover, governments have frequently failed to provide adequate public health communication to refugee groups [11, 12]. Although government advisory groups have been established to involve CALD leaders in pandemic responses [13], such bodies have not adequately included refugees [12]. By presenting culturally biased models of health information, governments risk reinforcing stigma and further isolating minority groups [14]. Stigma is associated with health services that are not culturally safe. According to Curtis et al. (2009), cultural safety requires healthcare professionals and organisations to examine themselves and the impact of their own culture on clients and service delivery [15]. Quality of care may be affected by biases, attitudes, assumptions, stereotypes, prejudices, structures and characteristics [15]. Thus, acknowledging and addressing these factors is the way forward for health services.

Despite the clear need for improved communication on COVID-19 to CALD populations, there is a relative dearth of literature on the subject. One qualitative Australian study conducted in 2020 found that CALD community organisations were effective intermediaries for public health communication, however this research was conducted with three large, well-established CALD groups in Melbourne (Chinese, Italian and Greek) [16]. The authors highlighted the need for future studies to focus on recent arrivals, including refugee communities [16]. Another Australian study conducted during the pandemic utilised a participatory approach to understand the role of CALD community leaders in communicating health information, however study participants were limited to community leaders, advocates and bi-cultural workers [17]. Other than one other study conducted by the authors during the pandemic [11], there is little evidence of refugee specific research related to COVID-19 message communication in Australia. Our previous research was conducted with a group of resettled Ezidi (or ‘Yazidi’) people in a different geographical location [11]. The study showed how refugee and cultural experiences had a significant impact on the sharing of COVID-19 messages in a tight-knit ethnic minority group [11].

There is an absence of understanding into how local newly emerging refugee communities respond to, receive and share COVID-19 messages. This current research explored aspects of these issues in a regional area of Australia, to identify ways to improve the communication of official COVID-19 information to these priority populations.

## Methods

### Aim

The aim of this study was to explore how culture, refugee experiences and existing relationships shaped what COVID-19 messages were listened to and shared. The work focused on three newly emerging refugee groups (Afghan, Congolese and Syrian) in a regional town in Australia.

### Design and setting

We undertook a qualitative study in the Hunter New England (HNE) region in New South Wales, Australia. The research team included staff from the local Multicultural Health Service and Population Health Unit. The study was approved by the Hunter New England Human Research Ethics Committee as low risk research; approval number: 2020/ETH02955. All research was performed in accordance with relevant guidelines and regulations of the Hunter New England Human Research Ethics Committee and Hunter New England Local Health District.

### Recruitment and participants

Participants were recruited by initial purposive sampling upon advice of the local Refugee Health (RH) nurse, with subsequent snowballing. Most community participants were invited by a member of the research team by telephone call, with the assistance of an interpreter. Service providers were invited by a member of the research team, either by email or telephone call. Service providers included representatives from settlement agencies, non-government assistance organisations and educational facilities who had had regular involvement with recently arrived refugee groups. All participants were over 18 years of age to ensure adequate consent was achieved. Community members were either Congolese, Afghan or Syrian background and had arrived recently in Australia on or after 1/1/2014; that is, 5 years preceding research commencement. Based on advice from service providers, researchers decided that this re-settlement period would represent peak interaction between newly arrived refugees and service providers and/or influential community members. These communities were chosen as they represented most recent refugee arrivals in the region, thereby forming newly emerging populations. Influential members were identified by the RH nurse as an individual who held a role of influence amongst either one or more of the communities. They included religious and ethnic leaders and cultural elders. Service providers included representatives from education facilities, settlement agencies and non-government organisations.

### Data collection and analysis

Semi-structured interviews were conducted by three staff members from the Multicultural and Refugee Health Service, who self-identified as being culturally and linguistically diverse: a Refugee Health Nurse, a Refugee Health Doctor and a Multicultural Health Liaison Officer. As only one interviewer had expert qualitative methods experience, prior to data collection, all three interviewers undertook a formal four-part qualitative training session provided by the co-author with doctoral qualifications in qualitative research methods (KB), focussing on interviewing techniques, discussion facilitation and equipment use.

Two group interviews (two participants and three participants in each) and one individual interview with service providers (March–April 2021); one group interview (three participants) and six individual interviews with influential members; and four group interviews (13 participants) and two individual interviews with community members (May–August 2021) were conducted. All interviews were conducted on-site at the local health service or participants’ homes, except for one influential member and one community member who were interviewed by telephone due to lock-down restrictions. Official interpreters attended interviews with limited-English speaking participants. Participant information was available as written English material or a video in Arabic, Swahili or Dari languages. The semi-structured interview guide included questions about sources and methods of COVID-19 message sharing among the community, associated challenges or facilitators, and suggestions for improvement. The guide was designed and piloted by the research team, prior to finalisation. All interviews lasted 30–60 minutes, were audio-recorded and transcribed verbatim in English by the lead researcher. Three layers of thematic analysis were employed: individual, paired and group [18]. Analysis was completed manually, without the use of software. Firstly, the two primary researchers read each written transcript separately, identifying key findings related to research questions. Next, the pair met regularly to reflect on their findings and to create categories, collating combined efforts into a new document. Categories were illustrated with participants’ de-identified quotes. The pair met three times with senior researchers to discuss categories and to identify overarching themes and subthemes.

## Results

Of the ten service providers invited, six agreed to participate. Of the 17 influential members invited, nine agreed to participate. Of the 24 community members invited, 15 agreed to participate. Two of the six service providers self-identified as also being influential community members. Five of the nine influential members self-identified as having had a refugee-like background themselves. Demographics of the community member participants are shown in Table 1.

**Table 1:** Demographic information of community participants, May–August 2021

Demographics		Afghan	Congolese	Syrian
Number of participants	Male	0	2	2
	Female	5	4	2
	**Total**	**5**	**6**	**4**
Age range	Age range (years)	25–40	18–50	40–50
Religion	Christian	0	2	0
	Muslim	5	4	4
Primary language spoken at home	Dari	2	–	–
	Hzargi	1	–	–
	Pashto	1	–	–
	Farsi	1	–	–
	Swahili	–	6	–
	Arabic	–	–	2
	Kurdish	–	–	2
Highest education achieved prior to resettlement	Nil to primary school	4	2	1
	Secondary school	1	3	2
	Tertiary education	0	1	1

We identified three major themes and several subthemes (Table 2). 1) Experience as a refugee uniquely influences COVID-19 message communication; 2) Refugee groups use diverse practices when accessing and sharing COVID-19 messages; 3) Official government messages could be improved by listening and tailoring to community needs.

**Table 2:** Themes and subthemes identified by thematic analysis of qualitative study

	Theme and subtheme
1	**Experience as a refugee uniquely influences COVID-19 message communication**
	• Educational background and English language proficiency
	• Mental health
	• Trust
	• Connectivity and social cohesion
	• Heterogeneity of culture, language and religion
2	**Refugee groups use diverse practices when accessing and sharing COVID-19 messages**
	• preferred sources of COVID-19 messages
	• ways of message sharing
3	**Official government messages could be improved by listening and tailoring to community needs**
	• Improved message content, delivery and format
	• greater cooperation and collaboration between communities and services
	• enhanced role of trusted service providers
	• research as a medium for mutual learning and community empowerment
	• supporting the messengers.

### Experience as a refugee uniquely influences COVID-19 message communication

Community members expressed how the ongoing lived experience as a refugee influenced access to COVID-19 health messages.

#### Educational background and English language proficiency

Community respondents described how fractured opportunities for education during times of war and escape led to reduced ability in general literacy. Furthermore, poor education coupled with low English proficiency contributed to feelings of inadequacy and low self-confidence. One Afghan community member explained that: *‘ … it’s really embarrassing if I go and ask....It’s like I’m saying I don’t have knowledge...’* A Congolese influential member reported that community members with lower English proficiency avoided attending COVID-19 information sessions because *‘ … they feel as if they are not good enough for that meeting, or not good enough to show themselves up … and speak there’.* A Syrian influential member observed that people in her community with lower levels of education were more likely to rely on others for information and respond to rumours than those who were educated to seek information for themselves.

Community members interpret the same English messages differently. One Afghan influential member said: *‘some people confused to be honest … most people English word have different meaning. Even I myself struggle with some of the words what exactly is that meaning and the content.’* Community members also reported that people had varying abilities in digital literacy, information and media literacy. Seeking out and determining accuracy of information was difficult to many in the community. One Afghan community member explained: *‘ … we have laptop- I don’t know what I should type, or what I should put. I don’t want to make mess or any mistake. So, I just leave it. And I’m not confident enough. And if I don’t know anything, I’m going to say “I don’t know it”. Just leave it...’* Another Afghan community member explained that: *‘Sometimes when you pass information from this person to the other, it’s like second hand news. You don’t know if it’s been altered or amended somewhere in between, and you don’t know how true it is.’*

On the other hand, influential and community members also reported that proficiency in English and baseline education were helpful in COVID-19 information seeking. Likewise, having access to an English-speaking family or friend was reported as beneficial for understanding health information provided in English. This was especially notable for Afghan community women, whose husbands had worked as interpreters for the Australian Defence Force.

#### Mental health

Some influential and community members also reported that anxiety and trauma, resulting from the refugee experience impacted interpretation and reactions to important information, such as COVID-19 messages. A Syrian influential member said: *‘Sometimes they [former refugees] may not get and understand the message sent to them … So sometimes we go to them, “okay, this is good for you, this is healthy”. Because sometimes, still they suffer from the trauma, and sometimes they may not take the [COVID-19] message in a straight way. Still they have, “we are still scared to get our citizenship … this [COVID-19] may be harmful for us”.’* Ongoing concerns about family overseas and the homeland COVID-19 situation were reported to contribute to panic and fear amongst communities. Apart from confusion and uncertainty, rumour was a source of anxiety and destabilisation, as one Syrian community member said: *‘Some people say the rumours and this make us like panic, get panic and get scared … we don’t like rumours. Rumours affects us.’* Furthermore, one Congolese influential member suggested that rumour might affect health seeking behaviour: *‘misinformation [rumour] itself is also making people not actually seek health advice if you’re ill because they are worried … [that] they might have Corona [virus-19] and that is a problem.’*

#### Trust

Service providers, influential and community members explained that trust was fundamental to relationship building and subsequently, was a major driver of message sharing. Community members reported great trust in their community leaders, as an Afghan community member reported: *‘ … the reason is that person being honest, and the past history shows that person did not do any mistake to put them under any question … and people they trust them, whatever things that they say.’* Similarly, a Congolese influential member reported that *‘community leaders are the actual people where multicultural people get information about COVID-19, because that’s their trusted system- their community leaders, their elders...’* Many community members also reported trusting their friends and family members to receive and understand COVID-19 messages, particularly those of their own sect who were educated or had proficiency in English. Community members described trusting known staff from service agencies with whom they had established relationships, such as: settlement agency case workers, refugee health nurses and English teachers. Settlement agency staff were particularly trusted by community members, described as having the means and experience to communicate trustworthy COVID-19 messages directly to community members. Some community members said they felt safe in Australia, having assurance of the health system and trust in official government messages.

#### Connectivity and social cohesion

Community members described how connectivity was vital for their new and emerging population, although at times, this was difficult during COVID-19 lockdown periods. A service provider reported that newly arrived refugees were limited in social connections due to the restrictions of lockdown and quarantine. Several community members agreed that connections to family, community and religious groups created a network for people to share information easily using their own language. A Congolese influential member commented on how connections were achieved: *‘We care about relationships. We reach out to these people … we mourn with them, we feast with them, we celebrate with them, we are really in their space.’*

Multilingual and educated community and influential members reported having a sense of social responsibility and obligation to connect and share important COVID-19 health information with others. Connections within family, such as school children who were fluent in English, helped translated messages to reach parents. Overseas connections to self-educate about COVID-19 were maintained by community members, by watching foreign television news and connecting through social media. Sometimes, being well-connected to overseas family, friends and media was noted to cause confusion and distress. A Congolese influential member explained: *‘So they just hear the news here [in Australia] and that side [overseas] … and then they [are] just more panicking. So I try to make [tell] them* “*… you need first to know what is happening here”’.* A Syrian influential member described how the social connection within refugee communities impacts message transmission: *‘they [former refugees] came from their country, it’s very social. [Here], they visit each other, and they help each other, and if someone knows an information, surely the others will get the information … ’.*

#### Heterogeneity of culture, language and religion

Some community members explained the inherent diversity within refugee groups, including languages, religious beliefs and cultural backgrounds. Many participants reported that official COVID-19 messages did not respond well to that diversity. For example, community and influential members explained that people of minority languages had limited access to official COVID-19 information. There was also variation in the preference of message tone- one Syrian influential member passed on messages in everyday friendly language; whilst another Syrian influential member said that the tone should be more formal, reporting that *‘when it’s friendly, they [community members] don’t follow it [message] up.’*

### Refugee groups use diverse practices when accessing and sharing COVID-19 messages

Participants indicated that past experiences of trauma and persecution, life opportunities and relationships all influence the sources and types of COVID-19 messages accessed by refugees.

#### Preferred sources of COVID-19 messages

Many community members noted that they preferred sources that had a simple message provided in language using a device that was easily accessible. Community members also preferred an audio-visual format, which did not contain text. An Afghan community member reported: *‘Reading news is really difficult. I always watch or listen. Listening and watching, because my reading is not as strong as others’.*

All groups agreed that the smartphone was a commonly used tool for community members to access COVID-19 related information. Smartphones provided access to numerous platforms such as social media, text messages, phone-calls, emails, government websites and applications. Community reported that social media (especially WhatsApp and Facebook), were commonly used to network with trusted friends and family, both locally and overseas. Social media updates and messages were also provided by trusted service providers and officials, such as religious groups, settlement agencies, English teachers and government health sector in addition to influential members. Many participants noted that social media was popular with refugee communities because the platform provided the opportunity to communicate with hundreds of contacts instantly and affordably. Social media platforms also provided the added option of relaying voice-messages, thereby appealing to those who were not confident with written language. Many community members also described how important the mobile phone was in sharing COVID-19 related information. A Congolese influential member reported: *‘ … it [telephone] was the only material available to use, whether you’ve been to school or not, or whether you’re rich or poor.’*

Some community members learnt about COVID-19 from pictorial signs or posters e.g., at shopping centres or other official organisations. Community members also reported trusting visual messages from overseas, especially television.

Some community members observed that settlement agencies were a useful and trusted source of COVID-19 information- particularly for newly arrived refugees who benefited from regular access to caseworkers providing active support and advice. Some community members also reported trusting information delivered by English language teachers and school teachers, staff from familiar non-government organisations and charities. Service providers and community members explained how Refugee Health staff delivered messages by telephone and face-to-face during medical consultations. All groups of participants, especially community members, praised Refugee Health staff as a trusted source of COVID-19 information. One Syrian influential member explained why this was so:

*‘when they [community members] have a message from the doctor or [RH nurse], they think it very serious issue. They feel it’s interference from the health professional, it’s something big, something massive happening … That is what they received back in Lebanon, or Jordan, and the orientation which they have from the United Nation [overseas] which they have, that ‘there will be a clinic called Refugee Clinic- it responsible for you there [in Australia]’.*

#### Ways of message sharing

Some community members reported accessing COVID-19 information opportunistically as opposed to intentionally. For example, some communities heard news from the radio or passengers whilst working as taxi or delivery (Uber) drivers. Some participants said that word of mouth was a common method of sharing information, for example during religious congregation, or with neighbours or friends attending English classes. One Afghan influential member explained that: *‘Any social gatherings, they share … they heard something, and it goes … viral after this’.* Some community members reported that face-to face presentation of information was easier for sharing messages, others preferred digital formats, as one Syrian community member reported: *‘Face to face is difficult, because some people are working, some people at home. But by message you can reach us … Even if they are at home, they can get it.’* Community members critiqued that lockdown inhibited opportunities for formal and informal sharing of COVID-19 news.

### Official government messages could be improved by listening and tailoring to community needs

Participants suggested various ways in which official information sharing about COVID-19 with refugee communities could be improved (Table 3). We describe them below in greater detail.

**Table 3:** Participant suggestions for better communication of COVID-19 messages to people of refugee backgrounds

• Use language/dialects preferred by communities	
• Use simple and clear messages	
• Provide regular updates	
• Present message visually, or by audio-link	
• Identify message as being from an official source	
• Harness social media	
• Use locations familiar to refugee communities to deliver face-to-face education (e.g., places of worship, non-government organisation hubs)	
• Deliver messages through trusted and familiar people	
• Be mindful of issues such as self-confidence and stress/trauma	
• Collaborate with influential members and service providers	
• Recognise diversity and avoid generalisation	
• Nurture community relationships with mutual respect	
• Enable mutual learning and community empowerment by continuing grass-roots research	

#### Improved message content, delivery and format

All three groups of participants suggested that official government messages could be improved by using a visual format in the appropriate language, providing outreach face-to-face demonstration sessions at familiar locations, and using trusted people and staff to distribute messages. Community and influential members added that regular updates and direct contact by telephone or in-person would be helpful for those with low written and digital literacy. Influential members and service providers suggested to simplify messages, pitching them to reach those at a basic education level, with accurate translation. As one service provider remarked: *‘if the English copy is academic and terrible, then the translation will be academic and terrible’.* One Afghan influential member summarised that: *‘I like the idea, when you post video [on social media] … in all languages. 1 minute, weekly, twice, something like that, it would be more helpful. And good thing is to pick people from communities itself. Religious leaders, doctors, GP [general practitioners], people know, and it will be good.’*

Some influential participants reported that governments need to mutually respect and acknowledge inherent diversity within communities, to provide acceptable COVID-19 messages. One Afghan influential member expressed the significance of respecting and recognising different sects:

*‘Let me tell little thing about Afghan- you know Afghan, we have different tribes, like hundreds different tribes. Each tribe, this is in an Afghan’s DNA, ok- “first respect me, then I respect you for my entire life”. If we came up with interpreter and doctor to interpret in Pashto and if I send that video to Pashto tribe, they would love it. They would say, “they are respecting us, they are interpreting in our own language”. Which means they feel respect. And also if we do the same thing in Dari, that would be nice as well.’*

#### Greater cooperation and collaboration between communities and services

Community members also noted that communication about COVID-19 could be improved if service providers, such as settlement agencies, government organisations and charities, worked collaboratively with community and their influential members to share accurate and up to date COVID-19 information. All participant groups recommended enhancing the role and response of Refugee Health and other public health organisations. Service providers and influential community members reported a desire to collaborate closer with government health services and had confidence that this would result in a better service for community. One Afghan influential member remarked:

*‘Look, this is a really nice work when people say ‘let’s work together’, and if we work together, we can pass this message to the community. You can pass it through me, through other community members, and that is really easy. I honestly do my job, I will share, and the community will take the benefit of that …*. *If you have 3 or 4 active members, who is connected with you guys, then I’m sure we would cover all the community with the accurate news and important news.’*

#### Enhanced role of trusted service providers

Influential members and service providers encouraged Refugee Health to extend their advocacy and research roles to assist communities. One Congolese influential member put it this way: *‘you [refugee health staff] are also dealing with the most difficult people that are even finding it hard to get the message by themselves … You guys are probably the perfect people in the centre of all this.’* One service provider suggested increasing the visual presence of the Refugee Health Team in promoting COVID-19 related educational material. Similarly, community members reported that information distributed by the settlement services would be well accepted, due to established trust.

#### Research as a medium for mutual learning and community empowerment

Some community members identified research as an opportunity to voice their experiences and improve current services. One Congolese influential member said: *‘ … doing something like this [research group], in a meeting, face to face, I think it’s sort of like, giving us also the chance of saying, we can do better’.* Another Congolese influential member suggested that: *‘One of the thing [problem] I think of is the lack of research. And those top government organisation how to work with these community members in order to spread the information’.* Some community members took the opportunity of the research interview to clarify COVID-19 information, by directly asking interviewers. One community member said: *‘Even you telling me about how far the COVID has gone, from sitting here, that can help me understand’.*

#### Supporting the messengers

Some service providers reported feeling unsupported by government agencies to deliver COVID-19 messages, particularly at the start of the pandemic. Influential members and service providers indicated that government services could consider a ‘train the trainers’ approach- providing official educational sessions to influential people who could distribute that message to the broader community. A Congolese influential member explained: *‘I would suggest training the trainers and also having people who will deliver those programs into the community. And knowing those communities who have the capability to deliver the message, it would be under that addendum’.* An Afghan influential member explained that governments can assist by improving communication with influential members by directly providing up to date information: *‘… they [community members] are asking me “what we should do about this, what we should do about this” and I’m sitting for hours and hours googling that. If I get that directly information from you guys [government health service], then I can send that video-link to them, “ok, see this” and it’s easy for me, easy for them’.*

## Discussion

We found that numerous factors affected how communities accessed, shared and understood COVID-19 information. These factors were intertwined with community’s lived experiences as refugees, social connections and networks unique to each group.

Our research indicates that communication of health messages to people with refugee backgrounds requires more than a clear, simple message delivered in language. We found that trusted relationships created webs of connectivity, which allowed community members to gather COVID-19 related information from various sources. The implications for this research are summarised in Table 3. Some findings may be applicable to settings in other developed nations where migration of CALD communities has similarly occurred.

In this study, the preferred channels for accessing COVID-19 messages were influenced by personal experiences such as educational background, English literacy, workplace opportunities and device accessibility. The breadth of responses from community members demonstrate that diversity must be acknowledged and explored when tailoring messages to CALD communities. The importance of appreciating diversity for the tailoring of public health communications and behavioural programs has recently also been recognised in Australia [17]. It is a timely reminder that Australia hosts a diverse population of CALD communities representing a wide range of social, religious, political and economic backgrounds [19]. A multipronged public health response must be considered to enhance communication across all communities so that risk can be reduced equitably.

In this study, trust and familiarity were key for communities seeking COVID-19 health information. Many CALD groups have collectivist cultures with a preference for interpersonal communication in respect to information seeking [20]. Interestingly, prior to the pandemic, a 2019 Australian systematic review found that refugees resorted to seeking health information from familiar sources, as a result of official health message communication being inefficient and unhelpful [21]. The systematic review found that by consulting familiar sources to increase knowledge, refugees reclaimed their sense of power and autonomy [21]. Although our participants largely agreed that official information can be improved, we did not find that this was the sole basis for seeking information from familiar sources. Rather, we found that cultural cohesiveness and strong interpersonal relationships drove interpersonal communication related to COVID-19 health information.

Community members in this study favoured the tools of social media messaging, text messaging and telephone calls as they were established, familiar and user friendly. It has been postulated that the reliance on social media for information sharing during the pandemic has been in part due to restrictions of social distancing [22]. Communication of official COVID-19 messages may be enhanced by optimising use of social media to share clear, accurate and trustworthy messages.

Our research also highlighted some worrisome implications of the refugee experience in relation to communicating health messages. First, participants discussed how education was related to self-confidence and thus engagement with the health sector. Poor literacy is a significant contributor to negative health outcomes, exacerbating health inequalities [23]. In addition, ineffective delivery of health information may result in disempowerment and loss of autonomy for refugees in Australia [19]. Secondly, participants noted that many refugees live with post-traumatic stress disorder (PTSD) and this may impact access to and sharing of health information. PTSD may affect memory and cognitive ability and executive functioning [24]. Similarly, PTSD symptoms may be aggravated by social isolation and empty streets caused by lockdowns, as they evoke memories of forced hiding [24]. The effect of post-traumatic stress disorder on COVID-19 message uptake is remarkable and corroborates findings from our previous research study with Ezidi refugees [11]. Importantly, we found no other published research on the impact of self-confidence or pre-existing trauma-related stress upon peoples’ responses and uptake of public health messaging. Further exploration of this insight would be worthwhile.

This study supports other research endorsing the role of community leaders and other influential and credible people in effectively delivering messages that are acceptable to, and trusted by community [17]. In a similar vein, we also found that religious leaders have a significant role for CALD communities in promoting desired behaviours [25]. Contrary to earlier findings, however [26–28], we found that many community members also trusted government sources of information. In particular, we found that the Refugee Health clinical staff played a powerful role in the perceived credibility of official government health messages. As with other service providers and community leaders, we attributed this to already established, trusted relationships.

Service providers who are trusted by the community can play an important role during public health responses [11, 29]. The research team, who included Refugee Health service providers, were encouraged by influential members and community members to continue collaborative efforts in research, education and advocacy to benefit the community. Collaborating with and listening to community provides opportunities for community voices to be heard and be empowered [30].

Community engagement has also been employed in the management of other infectious disease outbreaks such as Ebola [30]. It is known that involving community gives insight into community beliefs and practices, but there is often a gap in the implementation of these approaches by health providers [31]. Involving community also provides unique perspectives which can tailor policies to ensure they are fit for purpose [26]. Our study highlights the value of mutual respect when engaging with refugee communities, and further study in this arena for policy is recommended. Respectfully consulting communities and working together to tailor shared solutions to public health problems is the way forward [27, 30]. Continued engagement is necessary to foster trusting relationships between government and community, thereby solidifying connectedness and maximising success in public health endeavours [30, 32].

### Limitations

Our study was limited by relatively small numbers of participants and the selection of majority refugee groups in the local area. Due to funding limitations, refugees from ethnic minorities or minority languages were not included in the study, therefore potential responses from these equally important groups are unknown. There was no follow-up of the people who declined to attend for interview, thus further limiting the information we gathered. The notion of diversity precludes that it is entirely possible that other means, methods and reasons for communication preference might exist amongst these communities. Also, individual experiences may not reflect those of the community and should not be generalised to encompass all refugees, nor other refugee communities from similar ethnic backgrounds. However, the findings do provide a starting point for further research, which is clearly necessary in the field.

## Conclusions

Globally, the COVID-19 pandemic has disproportionately impacted CALD communities. Effective public health messaging to CALD communities will be critical in addressing inequities now and into the future, and requires more than quality content to reach audiences in a culturally acceptable way. This current study highlights that new and emerging refugee CALD communities have a variety of methods of sharing COVID-19 information. Individual experiences, opportunities and other factors such as trust and connectivity all influence how refugee communities access and understand official COVID-19 messages. Government services should be alert to potential nuances of new and emerging groups, such as issues around self-confidence and mental health, when developing COVID-19 communication strategies and materials. Mutual respect is an important factor when engaging with refugee communities. Ongoing collaboration and further research is warranted to delineate nuances, understand how mutual respect is cultivated and enable more informed, practical improvements in policy.

## Data Availability

All data analysed during this study are included in this published article.

## Abbreviations

COVID-19: Coronavirus disease of 2019
CALD: Culturally and linguistically diverse
HNE: Hunter New England
RH: Refugee Health
PTSD: Post traumatic stress disorder
GP: General Practitioner (local family doctor)
